# Thyroid Disfunction in Critically Ill COVID-19 Patients. Relationship with In-Hospital Mortality

**DOI:** 10.3390/jcm10215057

**Published:** 2021-10-29

**Authors:** María Antonieta Ballesteros Vizoso, Albert Figueras Castilla, Antonia Barceló, Joan Maria Raurich, Paula Argente del Castillo, Daniel Morell-García, Julio Velasco, Jon Pérez-Bárcena, Juan Antonio Llompart-Pou

**Affiliations:** 1 Servei d’Anàlisi Clíniques, Hospital Universitari Son Espases, 07120 Palma, Spain; maria.ballesteros@ssib.es (M.A.B.V.); antonia.barcelo@ssib.es (A.B.); paula.argentedelcastillo@ssib.es (P.A.d.C.); daniel.morell@ssib.es (D.M.-G.); 2 Servei de Medicina Intensiva, Hospital Universitari Son Espases, 07120 Palma, Spain; albert.figueras@ssib.es (A.F.C.); jmraurich@gmail.com (J.M.R.); julio.velasco@ssib.es (J.V.); juan.perez@ssib.es (J.P.-B.); 3 Institut d’Investigació Sanitària Illes Balears (IdISBa), 07120 Palma, Spain

**Keywords:** COVID-19, critically ill patients, thyroid hormones, intensive care unit, mortality

## Abstract

The incidence of thyroid disfunction has not been analyzed in critically ill COVID-19 patients. Our objective was to analyze the relationship of the thyroid profile and in-hospital mortality in critically ill COVID-19 patients. This was a prospective single-center study involving critically ill COVID-19 patients admitted to the ICU of a tertiary University Hospital. Thyroid hormones were measured through drawing blood samples from a central venous catheter at ICU admission and on the fifth day. A multiple logistic regression analysis was performed to analyze the variables associated with mortality. The ability of the different thyroid hormones to predict in-hospital mortality was evaluated by calculating the receiver operating characteristics (ROCs) and the area under the curve (AUC). A total of 78 patients were included in the study at ICU admission; 72 had their thyroid profile measured at day 5. In-hospital mortality reached 29.5%. Multiple logistic regression analysis showed that variables associated with mortality were age and prior beta-blocker therapy at ICU admission and age fT4 at day 5. The AUC for in-hospital mortality predictions of fT4 at day 5 was 0.69. Thyroid responses are commonly observed in critically ill COVID-19 patients. fT4 at day 5 after ICU admission was associated with mortality.

## 1. Introduction

The thyroid gland produces hormones that primarily regulate cellular metabolism through a wide range of pleiotropic effects which are essential for life. Although it is well-known that during general critical illness the most active form of thyroid hormone (triiodothyronine [fT3]) decreases, it remains controversial as to whether this is an adaptive response or a pathological condition which needs to be reversed [1]. 

In critical illness, thyroid hormone profiles are usually disturbed. The most typical alterations are low plasma concentrations of free triiodothyronine (fT3), low or normal plasma concentrations of free thyroxine (fT4), or elevated plasma rT3 in the presence of normal thyrotropin (TSH). Altogether, these alterations constitute so-called non-thyroidal illness syndrome (NTIS) [2,3]. Tissue concentrations of thyroid hormones seem proportional to circulating levels during critical illness [3]. 

The COVID-19 pandemic has challenged intensive care around the world [4]. In these patients, thyroid responses could also potentially be disturbed. Angiotensin-converting enzyme 2 (ACE2), the functional receptor for SARS-CoV-2, plays a role in the pathogenesis of COVID-19. ACE2 expression is present in many endocrine organs, including the endocrine pancreas and the thyroid gland [5,6]. To date, and from the clinical point of view, studies have been performed in heterogeneous populations of COVID-19 patients, and the relationship of the disease with outcomes remains unclear [7,8,9,10,11]. The incidence of thyroid dysfunction seems related to the severity of illness, but this has not been extensively addressed in critically ill COVID-19 patients [7,8,9,10,11].

Therefore, our objective was to analyze the relationship of the thyroid profile and in-hospital mortality in critically ill COVID-19 patients admitted to the intensive care unit (ICU).

## 2. Materials and Methods

This was a prospective, single-center study involving critically ill COVID-19 patients admitted to the ICU of a tertiary University Hospital (Hospital Universitari Son Espases, Palma, Spain). The ICU has 44 structural beds and usually admits medical, surgical and traumatic critically ill patients. The study was approved by the Comité Ètic Illes Balears (ref IB 4445/21 PI). Written informed consent was obtained from patients or closest relatives.

### 2.1. Patients

Consecutive adult (≥18 years) critically ill COVID-19 patients admitted to the ICU of our University Hospital from January to March 2021 were included. Infection was confirmed by means of reverse transcription–polymerase chain reaction (RT-PCR) from a nasopharyngeal swab or tracheal aspirate (if intubated). Patients were excluded if they had a prior history of thyroid disease and/or were on anti-thyroid drugs and/or thyroid hormone replacement. Notably, all patients received steroids as per the protocol for severe COVID-19.

### 2.2. Outcomes

The main outcome was to analyze the relationship of the thyroid profile and in-hospital mortality in critically ill COVID-19 patients.

### 2.3. Measurement of Thyroid Hormones 

Thyroid hormones were measured through drawing blood samples from a central venous catheter. Blood samples were collected the next morning after ICU admission and on the 5th day after ICU admission at 07:00.

Serum samples were collected in a serum separator tube and were centrifuged (3000 rpm, 10 min). After pretreatment of the sample and determination of the thyroid panel, two aliquots were frozen at −80 °C for possible future testing. Analyses of serum concentrations of TSH, fT4 and fT3 throughout the data collection process were performed on ARCHITECT i2000 (Abbott Diagnostics, USA). The ARCHITECT two-step immunoassay uses Chemiluminescent Microparticle Immunoassay (CMIA) technology. On the other hand, the analysis of reverse triiodothyronine (rT3) was measured by competitive radioimmunoassay (RIA). The manufacturer reports a linearity of 0.01–100 µUI/mL for TSH, 0.4–5 ng/dL for fT4 assays, 1.5–20 pg/mL for fT3 assays and 0.09–0.35 ng/mL for rT3 assays. Long-term inter-assay imprecision was satisfactory, with TSH coefficients of variation (CV) of 5.26% at 0.38 µUI/mL and 4.37% at 4.58 µUI/mL, fT4 CV of 3.6% at 0.98 pmol/L and 4.08% at 1.98 pmol/L, fT3 CV of 4.3 at 2.28 pg/mL and 5.12% at 6.05 pg/mL and rT3 CV of 7.4% at 0.28 ng/mL and 5.7% at 0.62 ng/mL. Lower limits of detection for TSH, fT4, fT3 and rT3 were <0.01 µUI/mL, 0.4 ng/dL, 1.5 pg/mL and 0.09 ng/mL, respectively. Reference values for TSH, fT4, fT3 and rT3 were 0.35–4.95 µUI/mL, 0.70–1.48 ng/dL, 1.58–3.91 pg/mL and 0.09–0.35 ng/mL, respectively. The laboratory maintenance was reviewed regularly according to manufacturers’ specifications. 

### 2.4. Statistical Analysis

Categorical data are expressed as numbers and percentages. Continuous variables are shown as the mean ± SD or median and interquartile range (IQR), as appropriate. Differences between groups were compared using the independent Student’s *t*-test and paired-samples *t*-test, or the Mann–Whitney U test (for continuous variables) and chi-squared or Fisher’s exact test (for categorical variables), as appropriate. A multiple logistic regression analysis was performed to analyze the variables associated with mortality in critically ill COVID-19 patients. The variables entered in the logistic regression analysis were those significantly associated with mortality in the univariate analysis. The ability of the different thyroid hormones to predict in-hospital mortality was evaluated by calculating the receiver operating characteristics (ROCs) and the area under the curve (AUC) and its 95% confidence interval (95% CI). *p* < 0.05 was considered statistically significant. Data were analyzed using the SPSS statistical package, version 22.0 (SPSS Inc., Chicago, IL, USA).

## 3. Results

During the study period, 84 critically ill COVID-19 patients were admitted to the ICU. Of them, six had previously had thyroid disease and were receiving treatment with thyroid hormone replacement; therefore, they were excluded from the study. Overall, 78 patients were included in the study at ICU admission, and 72 had their thyroid profile measured at day 5, because 6 patients died while in the ICU before obtaining the second sample.

Baseline clinical characteristics of the critically ill COVID-19 patients included in the study distributed by in-hospital mortality are shown in Table 1. No differences were found in terms of specific treatments received between survivors and non-survivors: remdesivir (16.4% vs. 4.3%, *p* = 0.27), tocilizumab (14.5% vs. 26.1%, *p* = 0.33) and prophylactic (54.5% vs. 56.5%), intermediate dose (36.4% vs. 34.8%) and systemic anticoagulation (9.1% vs. 8.7%), *p* = 1.0. As stated, all patients received steroids as per the protocol. No differences were found in terms of mechanical ventilation (80% vs. 95%) or extracorporeal life support (7.3% vs. 4.3%), *p* = 0.15. Days on mechanical ventilation were similar between survivors and non-survivors (median 18 (10–46) vs. 20 (5–36), *p* = 0.30). No differences were found in ICU length of stay (media 19 (10–49) vs. 17 (5–36), *p* = 0.2. In-hospital length of stay was higher in survivors (median 37 (22–83) vs. 18 (7–39), *p* = 0.002).

Similarly, survivors and non-survivors had similar rates of complications. Secondary infections: bacteriemia (30.9% vs. 39.1%, *p* = 0.60), nosocomial pneumonia (40.9% vs. 50%, *p* = 0.60), urinary tract infection (27.3% vs. 13%, *p* = 0.24). The incidence of acute kidney injury was higher in non-survivors (52.2% vs. 20%, *p* = 0.01). 

**Figure 1:**
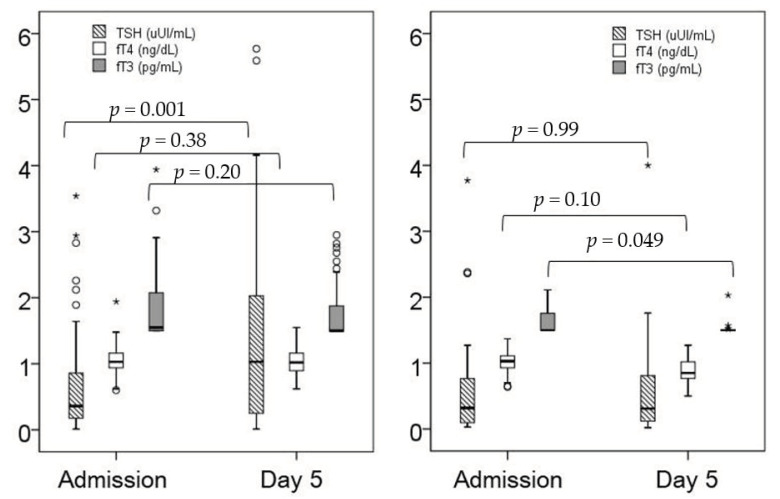
Changes in the thyroid profile over time in survivors (**left**) and non-survivors (**right**). *: *p*< 0.05.

At ICU admission, there were differences in the levels of fT3 between patients who survived and those who died (Table 2). On day 5 after ICU admission, we found differences both in fT3 and fT4 levels between survivors and critically ill COVID-19 patients who died (Table 2). No differences were found in the levels of reverse fT3 or in the incidence of NTIS between both groups (Table 2). Patients who survived had higher levels of fT3, fT4 and TSH than non-survivors by day 5 (Table 2). By day 5, survivors significantly increased their TSH levels (Figure 1). 

The multiple logistic regression analysis showed that variables associated with death in critically ill COVID-19 patients were age and prior beta-blocker therapy at ICU admission, and age and fT4 at day 5 after ICU admission (Table 3). 

**Figure 2:**
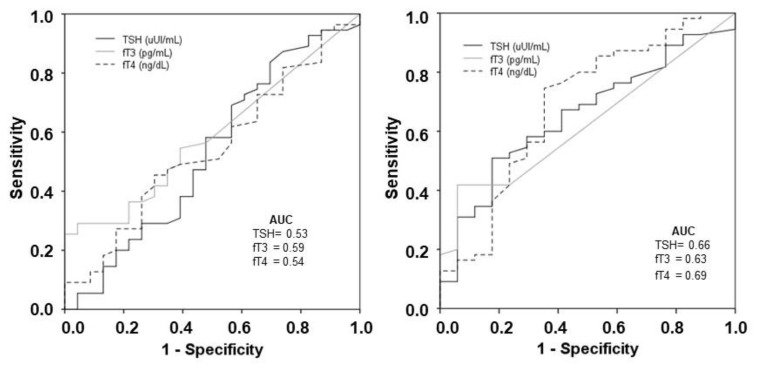
Receiver operating characteristic (ROC) curves of thyroid hormones at ICU admission and day 5 to predict in-hospital mortality.

The ability of the thyroid profile at ICU admission and day 5 to predict in-hospital mortality is moderate, as shown in Figure 2.

## 4. Discussion

In our population of critically ill COVID-19 patients, we found that thyroid response was commonly affected. In addition, fT4 at day 5 after ICU admission was associated with mortality. The incidence of NTIS was high in our group, but it was not associated with in-hospital mortality. 

fT4 is the most abundant thyroid hormone and is produced solely from the thyroid gland (approximately 100 mg per day). Plasma fT3 is derived largely from the extrathyroidal deiodination of fT4, and 20% is directly from thyroid secretion. Levels of thyroid hormones change during illness. Low fT3 is present in approximately half of patients at the time of admission to the ICU. A low fT4 level is found in 10% of patients at ICU admission, with up to 45% developing subnormal levels during their illness [1,12]. The extent of change in thyroid hormone measured at any time during ICU admission is proportional to the severity of illness, ICU length of stay and mortality rate [1,12,13]. The differences in the predictive effect of fT3 and fT4 on adverse outcomes in various studies may be related to differences in the etiological diagnosis and development period of severely ill patients in the ICU [9]. 

In our sample, we found that fT4 at day 5 after ICU admission was a risk factor for mortality in critically ill COVID-19 patients. Similarly to our study, Meyer et al. [14] showed that low fT4 levels during the course of the disease was a prognostic marker in critically ill patients, before the COVID-19 pandemic occurred. As in our results in critically ill COVID-19 patients, admission thyroid hormone levels were not prognostic in their cohort of critical patients. Peeters et al. [15], in a classical study of thyroid hormone changes during critical illness, enrolled 451 patients with thyroid hormones measured at days 1, 5, and 15 and the last day of their ICU admission, and described an increase in peripheral thyroid hormones in surviving patients, whereas thyroid hormones in patients who died decreased (15), similarly to what occurred in preliminary studies involving COVID-19 patients [16,17] This is in line with previous studies suggesting that an increase in TSH drives the rise in T4, and thus points to the onset of recovery [18,19]. In our study, TSH also rose in survivors from admission to day 5, and non-survivors presented a decrease in fT3 with no increase in TSH (Figure 1 and Table 2), supporting this hypothesis. Our patients were admitted in all cases because of severe COVID-19; therefore, our findings suggest that thyroid dynamics depend on the development period of severe illness rather than on the etiology. 

To date, studies in COVID-19 patients have shown that thyroid dysfunction seems related to the severity of illness [7,8,9,10,11]. In our sample of critically ill COVID-19 patients, low fT4 levels by day 5 were significantly associated with in-hospital mortality, although its predictive ability of in-hospital mortality was moderate. Whether thyroid hormone replacement in critically ill COVID-19 patients could potentially impact outcomes remains to be determined. According to our data, selecting which population could ultimately benefit from supplementation could not be determined by thyroid hormone levels at admission, making the challenge of replacement even more complex. Ongoing studies will help to elucidate the exact role of thyroid replacement in COVID-19 patients [20].

We must acknowledge the limitations of our study: first, we studied a relatively small sample size of critically ill COVID-19 patients. However, our population was severe and homogeneous, as indicated by the 84% needing invasive mechanical ventilation (the remaining 16% received oxygenation with a high-flow nasal cannula). Secondly, all patients included were receiving steroid treatment. This could have potentially affected the thyroid response [21]. However, because the same doses and steroid protocols were applied to all patients, we believe that this potential confounding factor was minimized. Thirdly, during the period when the study was performed, the predominant variant of the SARS-CoV-2 was B.1.1.7. Clinical manifestations may differ between different variants [22]; therefore, we cannot rule out that our findings may not be applicable to other variants. Lastly, the lower limit of detection, especially for fT3, was close to the mean values observed. This could potentially bias the interpretation of fT3 values.

## 5. Conclusions

We found that the thyroid response is commonly affected in critically ill COVID-19 patients. In addition, fT4 levels at day 5 after ICU admission were associated with mortality. The incidence of NTIS was high in our group, but it was not associated with in-hospital mortality.

## Figures and Tables

**Table 1:** Baseline clinical characteristics of the critically ill COVID-19 patients included in the study.

	Survivors N: 55	Non-Survivors N: 23	*p* Value
Female, n (%)	17 (30.9)	6 (26.1)	0.79
Age (years)	59 ± 12	68 ± 12	0.002
Weight (kg)	86 ± 20	80 ± 14	0.10
Height (cm)	170 ± 11	167 ± 10	0.27
Body mass index	29.8 ± 6.6	29.4 ± 5.1	0.74
Symptoms to sampling, days	10 (7–13)	9 (6–11)	0.58
SAPS II	38 (31–47)	42 (37–50)	0.01
APACHE II	17 (12–23)	21 (15–24)	0.08
Admission SOFA	5 (4–7)	5 (4–8)	0.33
**Comorbidities, n (%)**			
Hypertension	26 (47.3)	17 (73.9)	0.04
Diabetes mellitus	12 (21.8)	8 (34.8)	0.26
Dyslipidemia	23 (41.8)	14 (60.9)	0.14
COPD	6 (10.9)	4 (17.4)	0.47
Chronic kidney disease	4 (7.3)	5 (21.7)	0.11
Chronic liver disease	1 (1.8)	3 (13.0)	0.07
Chronic cardiac disease	4 (7.3)	8 (34.8)	0.004
Cancer (active)	5 (9.1)	4 (17.4)	0.44
**Prior treatment, n (%)**			
Beta-blockers	5 (9.1)	8 (34.8)	0.02
ACE inhibitors	9 (16.4)	8 (34.8)	0.13
ARBs	12 (21.8)	7 (30.4)	0.56
Statins	18 (32.7)	12 (52.2)	0.13

SAPS II: Simplified Acute Physiology Score II; APACHE II: Acute Physiology and Chronic Health Evaluation II; SOFA: Sepsis-related Organ Failure Assessment; COPD: chronic obstructive pulmonary disease; ACE: angiotensin-converting enzyme; ARBs: angiotensin receptor blockers.

**Table 2:** Comparison of thyroid profile at ICU admission and day 5.

	Survivors	Non-Survivors	*p* Value
** Thyroid profile ICU admission **	**n: 55**	**n: 23**	
TSH, uUI/mL	0.68 ± 0.79	0.70 ± 0.94	0.91
fT3, pg/mL *	1.8 ± 0.5	1.6 ± 0.2	0.02
fT4, ng/dL	1.1 ± 0.2	1.0 ± 0.2	0.40
Reverse fT3, ng/mL	0.69 ± 0.23	0.65 ± 0.26	0.64
NTIS, n (%)	24 (43.6)	12 (52.2)	0.62
** Thyroid profile day 5 **	**n: 55**	**n: 17**	
TSH, uUI/mL	1.46 ± 1.69	0.70 ± 0.99	0.08
fT3, pg/mL *	1.8 ± 0.4	1.5 ± 0.1	0.001
fT4, ng/dL	1.0 ± 0.2	0.9 ± 0.2	0.02
Reverse fT3, ng/mL	0.49 ± 0.24	0.38 ± 0.17	0.19
NTIS, n (%)	30 (54.5)	13 (56.5)	1.0

* Values below the lower limit of detection in some patients. *NTIS:* non-thyroidal illness syndrome.

**Table 3:** Variables associated with death in critically ill COVID-19 patients.

	OR	95% CI	*p* Value
**ICU admission**			
Age	1.07	1.02 to 1.14	0.01
Beta-blocker therapy	4.25	1.15 to 15.76	0.03
**Day 5**			
Age	1.09	1.02 to 1.17	0.01
fT4	0.02	0.001 to 0.53	0.02

## Data Availability

Data used in this study can be obtained from the corresponding author upon reasonable request.
